# Comparison of Different Algorithms for Calculating Velocity and Stride Length in Running Using Inertial Measurement Units

**DOI:** 10.3390/s18124194

**Published:** 2018-11-30

**Authors:** Markus Zrenner, Stefan Gradl, Ulf Jensen, Martin Ullrich, Bjoern M. Eskofier

**Affiliations:** 1 Machine Learning and Data Analytics Lab, Department of Computer Science, Friedrich-Alexander-Universität Erlangen-Nürnberg (FAU), 91052 Erlangen, Germany; stefan.gradl@fau.de (S.G.); martin.ullrich@fau.de (M.U.); bjoern.eskofier@fau.de (B.M.E.); 2 Finance & IT—IT Innovation, Adidas AG, 91074 Herzogenaurach, Germany; ulf.jensen@adidas.com

**Keywords:** wearable sensors, inertial measurement unit, gait, running, stride length, velocity, smart shoe

## Abstract

Running has a positive impact on human health and is an accessible sport for most people. There is high demand for tracking running performance and progress for amateurs and professionals alike. The parameters velocity and distance are thereby of main interest. In this work, we evaluate the accuracy of four algorithms, which calculate the stride velocity and stride length during running using data of an inertial measurement unit (IMU) placed in the midsole of a running shoe. The four algorithms are based on stride time, foot acceleration, foot trajectory estimation, and deep learning, respectively. They are compared using two studies: a laboratory-based study comprising 2377 strides from 27 subjects with 3D motion tracking as a reference and a field study comprising 12 subjects performing a 3.2-km run in a real-world setup. The results show that the foot trajectory estimation algorithm performs best, achieving a mean error of 0.032 ± 0.274 m/s for the velocity estimation and 0.022 ± 0.157 m for the stride length. An interesting alternative for systems with a low energy budget is the acceleration-based approach. Our results support the implementation decision for running velocity and distance tracking using IMUs embedded in the sole of a running shoe.

## 1. Introduction

Distance running is a very popular sport. Two main reasons for this popularity are simplicity and the health benefit, as running can be done in small and restricted time-frames and does not require a specific location. Besides sports gear, no equipment is needed. Moreover, running improves health. Studies have shown that aerobic endurance training like running can reduce blood pressure [1] and that moderate running twice a week (>51 min or six miles) reduces overall mortality risk and the occurrence of cardiovascular diseases [2]. However, overtraining can also lead to a higher risk of injury of the lower extremities for distance runners [3].

Tracking running performance over time can prevent overtraining and greatly support a healthy and effective training. A training diary helps to maintain the right training intensity and volume, which are essential for both performance and health enhancement. Training records are also motivating, as they highlight both effort and progress. However, a precise, objective, and easy measurement of relevant parameters is needed. Two common parameters that both professional and amateur runners use to track their performance is the average velocity and total distance. With these parameters, the running workout can be easily categorized, rated, and compared. In the past, runners estimated the distance of a predefined running track and took time with a stopwatch to calculate the average velocity of a distance run. With the rise of wearable technology in recent years, easier and more precise methods have become available.

### 1.1. Literature Review

The predominant approach to tracking average velocity and total distance during running is the global positioning system (GPS). Smartphones or even smartwatches comprise a GPS chip, which allows a satellite-based localization of a runner. By tracking the runner’s absolute position over a complete run and using a solution to the second geodetic problem [4], the distance of a run can be measured. By incorporating the sampling frequency of the GPS module, a continuous time series of velocity values for the run can be computed. Thus, GPS delivers a time series of velocity, the cumulative distance, and the localization of the running track. From these data, the average velocity and the total distance can be extracted. The drawbacks of GPS are the additional gear (smartwatch, smartphone), the high energy demand, and the restriction to outdoor use.

Integrating sensors directly into running shoes can solve these issues. One type of sensor that can be integrated into a shoe is an inertial measurement unit (IMU). It is a small, lightweight, and inexpensive sensor, which is capable of measuring triaxial accelerations and triaxial angular rates. A shoe setup with integrated IMUs overcomes the described GPS issues: runners only need a running shoe with integrated IMU; IMUs are energy efficient and work both indoors and outdoors. Using IMU data, it is possible to compute a stride length and an average velocity value per stride. The underlying assumption for the velocity computation is that the average velocity of the foot per stride matches the running velocity. By collecting stride velocity values and accumulating the stride length values over time, a distance measure and a continuous velocity recording of a complete run can be provided. The following paragraphs describe the state-of-the-art of four approaches for IMU data processing for calculating these metrics.

In biomechanics, the relationships between stride frequency, stride length, running velocity, and body height was investigated [5]. The results indicated that with increasing running velocity, stride frequency and stride length increase. Thus, increasing running velocity is an interaction of increasing stride length and stride frequency [5]. Stride length itself depends on body height and can be expressed as a relative stride length. From these relationships, a generic model relating running velocity and stride length on the basis of the stride frequency can be deduced. The general idea behind this approach is the inverse correlation between velocity and stride time (the higher the velocity, the shorter the stride time). Thus, in order to estimate the stride length, only the stride time has to be distinguished by segmenting the data into single strides. An average velocity of the stride can then be calculated using the stride length and the measured stride duration.

Recently, Gradl et al. [6] proposed an algorithm that uses quadratic regression to compute the velocity of movements. The velocity was evaluated during running, as well as other movements and showed a relative error of 6.9±5.5%. The proposed algorithm is solely based on foot acceleration. Single strides are segmented from the data stream. Afterwards, the acceleration signal of all axes is integrated prior to the initial ground contact. Finally, the resulting integral value is converted to a velocity value using a quadratic regression model.

Another method to compute velocity and stride length values from IMU signals is to reconstruct the trajectory of the sensor in the course of a stride. This method is heavily used for gait analysis for geriatric patients [7,8,9] or in inertial navigation scenarios [10,11]. For trajectory reconstruction, sensor fusion techniques must be applied to both the accelerometer and the gyroscope. Several fusion algorithms to cope with this task exist. Bailey et al. [12] and Foxlin et al. [13] used extended Kalman filters to compute the trajectory from the acceleration and angular rate signals, while Rampp et al. [7] applied a linear dedrifting technique. Both algorithms rely on a zero-velocity update during the stance phase for the initialization of the orientation. The literature shows that this approach works well while analyzing walking [7], but it was not evaluated for free running. Bailey et al. [12] applied their approach to treadmill running and showed a good accuracy of 0.03±0.2 m/s. However, they evaluated neither the velocity nor the stride length in a free running scenario.

Deep learning techniques also show good results in IMU-based classification and regression tasks [14,15]. Hannink et al. [16] showed that deep convolutional neural network regression outperforms traditional stride length estimation in geriatric gait analysis. They trained a network with two convolutional layers, which was fed with the 6D IMU raw data of a stride. The output layer had a single node and provided an estimate of stride length.

### 1.2. Contribution

Most of the described algorithms were evaluated either for walking or for running on a treadmill. However, both of these conditions yield different signal characteristics to those of free running. In running, different strike patterns, such as rearfoot or forefoot strike, exist and affect the performance of these algorithms. Besides, the movement is also more dynamic, which yields higher accelerations, angular rates, and impacts. Therefore, our contribution is the comparison of different algorithmic approaches for computing average velocity and stride length during overground running using an IMU embedded into the sole of a running shoe. We evaluate these algorithms on a large database including high variation of the input data. Additionally, we run a field study to assess the performance in a real-world scenario. Based on the results, we give implementation recommendations for specific use cases.

## 2. Methods

### 2.1. Data Collection

We conducted two data collection studies for algorithm comparison, a lab study and a field study. The lab study was conducted in a sports research lab to evaluate the performance of the algorithms against ground-truth stride length and velocity labels on a per stride basis. A 3D motion tracking system was used as a reference. The field study was conducted on a 400-m outdoor running track to evaluate the performance regarding the total distance on a continuous 3.2-km run in realistic free-running conditions. The track length was used as a reference.

#### 2.1.1. Lab Study

In the lab study, data from 27 amateur runners (21 male, 6 female) were recorded. The dataset included runners with different strike types. Six of the subjects were forefoot/midfoot runners, and 21 subjects were rearfoot runners. The classification of the strike type was based on the definitions of Altman et al. [17]. Further anthropometric data can be found in Table 1. Before data acquisition, all subjects were informed about the related risks and gave written consent to participate in the study and for the collected data to be published.

**Figure 1:**
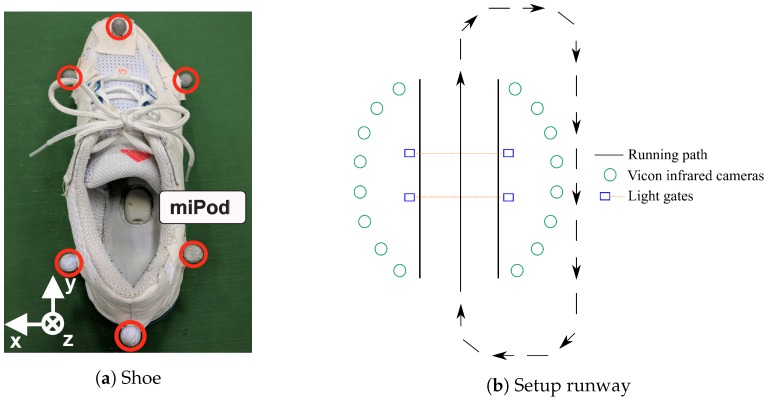
(**a**) Shoe equipped with a miPod sensor and the marker setup. The IMU is located within the sole of the running shoe. The marker setup allowed for a computation of velocity and stride length. (**b**) Illustration of reference system setup. The subjects ran through the capture volume of the motion tracking system, created by 16 infrared cameras, and looped back around.

The subjects were equipped with running shoes in matching sizes (Response Cushion 21, Adidas AG, Herzogenaurach, Germany), as depicted in Figure 1a. This model had a cavity in the right shoe midsole for the placement of a sensor. We cut another cavity of the same size at the same location into the left shoe midsole to be able to acquire data from both the left and the right shoe in order to record more data for the training and evaluation of the algorithms. The specific IMU we used was the miPod sensor [18]. The accelerometer of the sensor was configured with a range of ±16 g and the gyroscope with a range of ±2000 $\frac{\circ }{s}$, and data were sampled with a frequency of $f_{s}=200$ Hz and a resolution of 16 bit. Before each data acquisition, the IMUs were calibrated using the calibration procedure introduced by Ferraris et al. [19]. Figure 1a depicts the orientation of the sensor in the sole of the running shoe: *x* points in the lateral direction, *y* in the dorsoventral direction, and *z* in the craniocaudal direction.

As the gold standard for velocity and stride length, we used a motion capture system (Vicon Motion Systems Inc., Oxford, UK) with 16 infrared cameras and recorded data with a sampling rate of $f_{s}=200$ Hz. A submodel of the marker setup introduced by Michel et al. [20] containing six markers on each shoe (see Figure 1a) was used. The marker on the heel (for rearfoot runners) and the lateral sided toe marker (for forefoot/midfoot runners) were used to extract strides. Depending on the strike type, minima in the trajectory of the corresponding markers were used to label initial ground contacts [21]. The IMUs and the motion tracking system were synchronized using a wireless trigger [22], which was connected to light barriers (S40 Series, Datalogic, Bologna, Italy). The light barriers triggered the start and the end of the recording for each trial in both systems. Using the described synchronization technique, we were able to match strides in the motion capture gold standard data to strides in the IMU signal.

The subjects were asked to run various trials with different velocities in the range of 2–6 m/s. We defined these velocity ranges to cover a wide range of relevant running velocities. As the capture volume was restricted to 6 m, and the stride length varied depending on the running velocity, different numbers of strides were recorded for the different running velocities. We recorded five additional trials for the two high velocity ranges to increase the number of captured strides. The velocity ranges and number of trials recorded can be found in Table 2. The subjects were asked to accelerate before and keep the pace within the capture volume. We measured the velocity at the beginning of the motion capture system volume using the above-mentioned light barriers used for synchronization. The velocity measured by the light barriers was used to ensure that a sufficient number of trials were recorded within each velocity range, for each subject. If necessary, the subjects were instructed to run faster or slower in order to ensure the defined number of trials in each velocity range. The ground truth value for each stride’s velocity $v_{ref}$ was computed from the motion capture reference as:

(1) $$v_{ref}=\frac{d_{ref}}{t_{ref}}=\frac{d_{ref}\cdot f_{s}}{N_{stride}}$$

where $d_{ref}$ is the stride length obtained by the difference of the positional data obtained by the motion capture system between two consecutive initial ground contacts, $t_{ref}$ the corresponding reference stride time, $N_{stride}$ the number of samples in between two consecutive initial ground contacts, and $f_{s}$ the sampling rate. Figure 1b illustrates the setup and running path of the subjects during the lab data recording. Overall, 2377 strides were recorded during the lab study for the evaluation of the algorithms.

#### 2.1.2. Field Study

The goal of the field study was to evaluate the algorithm performance regarding total distance in a real-world scenario. We recorded twelve subjects who performed a self-paced 3.2-km run by completing eight rounds on a 400-m tartan track. We used this setup to be able to obtain a reference distance accurately.

The equipment (IMUs, shoes) and settings were the same as described in the lab study (Section 2.1.1) to enable a direct comparison of the results. The subjects participating in the field study were not part of the lab study. Additionally, we recorded GPS data using a smartphone (Galaxy S8, Samsung Inc., Seoul, South Korea) and the fitness application Strava (Strava, Strava Inc., San Francisco, CA, USA), which is, with 136 million uploaded runs in 2017, one of the most popular fitness apps worldwide [23]. It also has the capability to export the GPS track in the GPX-format [24] allowing for a computation of the distance of the running track. The accuracy of the implemented algorithms can be compared to the GPS data for the total distance of the run. We used the great circle distance to compute the total distance of the GPS measurements [25]. Our computed total distance from the exported GPX file matched the distance that Strava provided via its services. Thus, we could compare the accuracy of the different algorithms to state-of-the-art running platform distance measurements.

### 2.2. Algorithms

In this section, the algorithms will be described in detail. The section starts with the stride segmentation algorithm, which is required for all algorithms, except the acceleration-based algorithm, which includes a different approach to segment steps. Afterwards, the algorithms are described in the following order: *Stride time*, (foot) *Acceleration*, (foot) *Trajectory* estimation, and *Deep Learning*.

#### 2.2.1. Stride Segmentation

The first step in the IMU signal processing for velocity and distance calculation was the stride segmentation. In this step, single strides were extracted from the continuous IMU data stream with a threshold-based algorithm. Common algorithms use the distinct peaks in the acceleration signal in the dorsoventral direction $a_{y}\left[n\right]$ during initial ground contact to mark the beginning of a stride [26]. We enhanced this idea and used the beginning of the distinctive peak to mark the beginning of a stride. This procedure is valid, as the ground already exerts a force to the IMU at the time instance of the peak in the acceleration signal. Using the peak itself would mean to mark a point in time that is part of the ground contact. To find the sample before the acceleration peak, we first differentiated the acceleration signal in the dorsoventral direction $a_{y}\left[n\right]$ and consecutively squared the resulting value to obtain a signal H[n] with amplified peak values.

(2) $$H\left[n\right]={\left(a_{y}\left[n\right]-a_{y}\left[n-1\right]\right)}^{2}$$

In the signal H[n], the maxima were detected by comparing them to an empirical threshold (empirical threshold: $H\left[n\right]>1000\left[{\left(\frac{m}{s^{2}}\right)}^{2}\right]$). For every detected maximum, the onset of the rise of H[n] was determined by setting all values below the threshold to zero and looking for the index of the last non-zero value in H[n] before the detected maximum. This index $n_{IC}$ was a potential candidate for an initial ground contact. To eliminate false detections, we added a detector for the swing phase prior to the peak in the acceleration signal. The swing phase detector computed an integral of $a_{y}\left[n\right]$ backwards from the first detected non-zero value until the first zero-crossing. This is the point in time where the foot starts decelerating during the swing phase. The integral value $S[n_{IC}]$ for the initial ground contact candidate $n_{IC}$ was computed as:

(3) $$S\left[n_{IC}\right]=\sum_{n=n_{ZC}}^{n_{IC}}\frac{1}{f_{s}}a_{y}\left[n\right]$$

**Figure 2:**
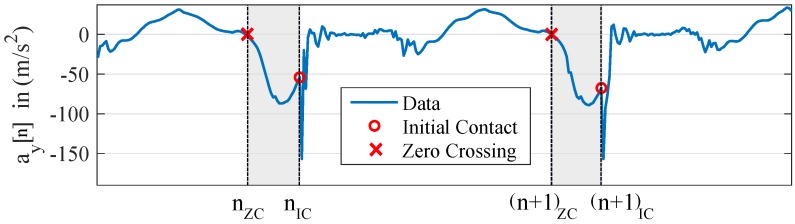
Example for the stride segmentation. The plot shows the acceleration signal in the dorsoventral direction $a_{y}\left[n\right]$, the detected initial ground contact $n_{IC}$, and the beginning of the swing phase (zero crossing $n_{ZC}$) to confirm the stride candidate. The marked area depicts the integration area for the swing phase detection.

In this equation, $n_{ZC}$ corresponds to the index of the zero crossing marking the start of the deceleration. If the integral value $S[n_{IC}]$ exceeds an empirically-set threshold (empirical threshold: $S\left[n_{IC}\right]<-3\left[\frac{m}{s}\right]$), a swing phase is detected, and thus, the index of the first non-zero value before the acceleration peak is labeled as an initial ground contact. The described stride segmentation is depicted in Figure 2.

#### 2.2.2. Stride Time

Cavanagh et al. described the relationship between running velocity, stride length, and stride frequency [5]. Stride frequency is an inverse measure of the stride time and describes the number of strides per minute. They showed that runners can increase their running velocity either by increasing their stride length or by increasing their stride frequency, thus decreasing the stride time. For lower velocities, runners tend to increase the stride length, while for higher velocities, they tend to increase the stride frequency. Thus, both the stride time and stride length have no linear dependency on running velocity. Furthermore, it has to be noted that runners control their velocity individually. The stride length and therefore the velocity also depend on other parameters like the gender and the height of the runner. Male runners show greater stride lengths compared to female runners. The stride length increases with the body height [5].

We used these biomechanical relations to build an algorithm that estimates stride length and velocity. Cavanagh et al. [5] provided averaged values for the non-linear correlation between the stride time $t_{stride}$ and a relative stride length $d_{stride,rel}$, which is calculated by dividing the absolute stride length $d_{stride}$ by the runner’s height *h*. We looked for further publications describing this relationship and came up with two step functions for males and females that discretized the underlying non-linear relationship between stride time and stride length for each gender. The definition of the functions for both male and female runners can be found in Table 3.

The stride time $t_{stride}$ was obtained by dividing the number of samples of one stride $N_{stride}$ by the sampling frequency $f_{s}$.

(4) $$t_{stride}=\frac{N_{stride}}{f_{s}}=\frac{{\left(n+1\right)}_{IC}-n_{IC}}{f_{s}}$$

$N_{stride}$ was computed by subtracting the indices of two consecutive initial ground contacts ${\left(n+1\right)}_{IC}$ and $n_{IC}$ obtained from the stride segmentation algorithm. After obtaining the relative stride length $d_{stride,rel}$ of the runner based on the gender and the stride time, the absolute stride length $d_{stride}$ was computed by multiplying $d_{stride,rel}$ from the table and the runner’s height *h* in meters.

(5) $$d_{stride}=h\cdot d_{stride,rel}$$

The running velocity $v_{stride}$ was then calculated using the stride time and the stride length.

(6) $$v_{stride}=\frac{d_{stride}}{t_{stride}}$$

Thus, the *Stride time* algorithm is solely based on the stride time. Gender and body height are usually known in all applications.

#### 2.2.3. Acceleration

The *Acceleration* method introduced in [6] uses only acceleration data for step segmentation and the computation of stride length and stride velocity. The method correlates the velocity of the foot (and thus, the subject) with the acceleration during the swing phase of the foot. It consists of three different algorithmic steps: (1) a continuous calculation of an integration value with a strong correlation to the movement velocity, (2) a stride segmentation based on initial ground contacts to determine the swing phase of the foot, and (3) a regression model to translate the continuous integration value to the velocity value.

We used the step segmentation algorithm from the cited publication due its applicability to movements other than running [6]. The inputs to the processing pipeline were the sampled triaxial acceleration signals from the foot sensor $a_{x}$, $a_{y}$, and $a_{z}$. After smoothing the input signals using a sliding window filter, the integration value ι was calculated as a multi-step absolute averaging across all directional components with:

(7) $$\iota \left[n\right]=\frac{1}{L+1}\sum_{i=0}^{L}\sum_{d=x,y,z}\left|s_{d}\left[n-i\right]\right|,$$

where *L* is the window length, which is expected to be the same as the duration of the foot swing phase.

**Figure 3:**
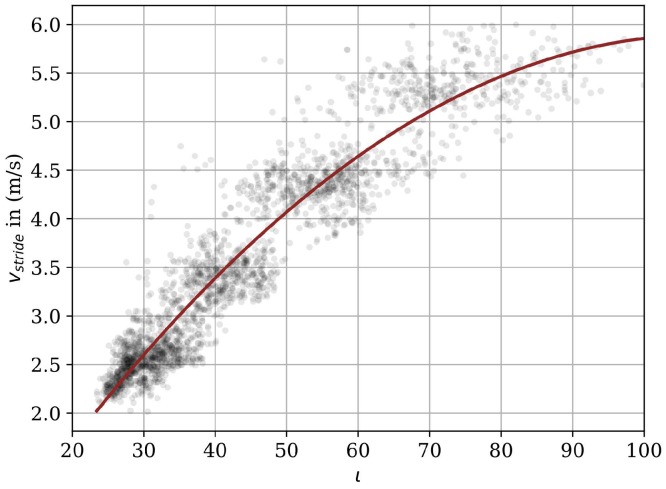
Polynomial function of second degree (red line) that relates the velocity integration value ι to the reference velocity values $v_{stride}$ (grey dots).

Individual strides were determined in the smoothed dorsoventral acceleration signal $s_{y}$ using a peak detection process in combination with two knowledge-based parameter thresholds. The goal was to detect and isolate the high impact response of initial ground contact in the smoothed signal [6]. Each time a valid stride was detected by the stride segmentation algorithm, the average velocity per stride $v_{stride}$ was determined based on a second degree polynomial regression function:

(8) $$v_{stride}=A+B\cdot \iota \left[n_{IC}\right]+C\cdot \iota {\left[n_{IC}\right]}^{2},$$

where the constants A,B, and *C* are derived during a regression model training phase where known reference velocity observations are matched to velocity integration values using parametric regression analysis. A trained regression model can be observed in Figure 3.

#### 2.2.4. Trajectory

Based on the foot trajectory, the stride length and stride velocity can be deduced. The trajectory of the sensor during running can be computed using an extended Kalman filter approach or using dedrifting techniques. We applied dedrifting techniques due to two reasons: Firstly, Bailey et al. [12] showed that the results for the mean step velocity of the two techniques did not differ significantly with respect to accuracy (extended Kalman filter: 0.03 ± 0.02 m/s, linear dedrifting: 0.0 ± 0.03 m/s). Secondly, the same authors showed in a different article that a sampling rate of more than 250 Hz is required for an extended Kalman filter approach [31]. For embedded use cases (e.g., a smart shoe scenario), low sampling rates are beneficial from an energy perspective. In gait analysis, the linear dedrifting technique showed promising results for a lower sampling rate of 200 Hz [7].

**Figure 4:**
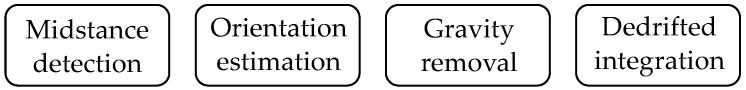
The four steps of the algorithm for the trajectory reconstruction based on linear dedrifting.

Trajectory reconstruction algorithms based on linear dedrifting consist of four steps, as depicted in Figure 4, and have both the triaxial accelerometer and the triaxial gyroscope measurements as an input. In the following paragraphs, the four algorithmic steps will be explained in detail. Orientation is computed by integrating the gyroscope measurements, and the position is obtained by integrating the accelerometer measurements.

**Midstance detection**: A common problem with computing the trajectory from IMU measurements is the drift of the sensors introduced by noise in the acceleration and the angular rate measurements. This drift is limited by using zero velocity updates [32]. The idea behind these updates is to reinitialize the position and the orientation of the sensor for every stride. By applying that technique, absolute position in space is lost; however, the individual stride parameters can be computed more accurately. The reason for the higher accuracy lies in the integration of shorter durations and thus a smaller accumulated error. The point in time for the reinitialization of the stride values originates from gait analysis and is the midstance phase during a stride cycle. At this point in time, the foot has its lowest velocity, and the orientation of the foot is known, because during midstance in gait, the foot is expected to be flat on the ground. Thus, it can be assumed that the orientation of the sensor can be computed statically using the acceleration measurement. This allows the initialization of the position and velocity to zero and the orientation with respect to gravity. To find midstance, we computed the minimum gyroscopic energy after initial ground contact [32] in a 250-ms time interval. The duration of this time interval is the average time of the stance phase for velocities up to 6 m/s [33]. Hereafter, the trajectory reconstruction will be performed on strides segmented from midstance to midstance.

**Orientation estimation**: After initializing the orientation based on the accelerometer measurement during midstance, the orientation of the sensor was computed using the gyroscope measurements. This step is necessary to calculate the orientation of the sensor so that gravity can be removed, which is an essential step for the computation of the position in space from the acceleration signal. For the orientation computation, we used the same quaternion integration approach as described by Rampp et al. [7].

**Gravity removal**: After the orientation estimation, gravity was removed. Without this removal, the gravitational acceleration of 9.81 m/s${}^{2}$ would be integrated additionally into the acceleration caused by running, which would lead to a large error over the duration of a stride. To remove gravity, we used the orientation of the sensor obtained by the gyroscope integration to rotate the acceleration measured in the sensor coordinate system to the world coordinate system. In the world coordinate system, we subtracted the gravitational acceleration from the measured acceleration.

**Figure 5:**
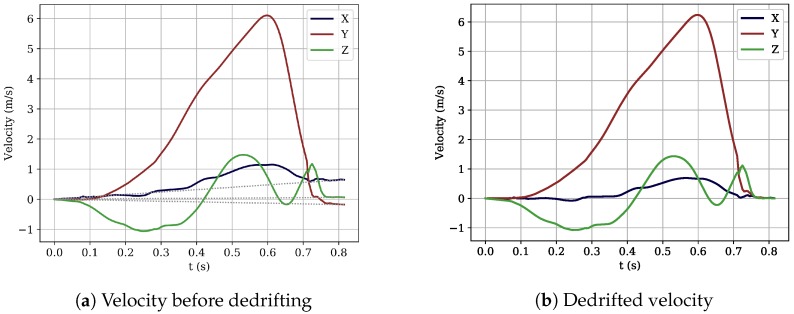
Visualization of the dedrifting method that ensures that the velocity during the second midstance is zero. (**a**) Velocity signal before dedrifting. The grey doted linear function is fit between the first and last point of the stride (midstance). (**b**) Velocity signal after dedrifting.

**Dedrifted integration**: The last step to come up with the full trajectory of the stride was to compute the position of the sensor by a double integration of the gravity removed acceleration. The first integration computed the velocity of the sensor over time, followed by the second integration, which resulted in the position of the sensor over time. Despite the gravity removal, there was still noise in the acceleration signal, causing drift in the results. This drift was reduced by dedrifting the velocity signal obtained after the first integration. The core idea behind dedrifting is the fact that we assume the velocity to be zero during midstance. For every stride, we fit a linear function in the velocity signal for all three directions, which was determined by the first and last velocity value of the stride. To dedrift the velocity signal, we subtracted the linear function from the integrated velocity signal, which enforced the velocity to be zero for both the first and the second midstance. This process is depicted in Figure 5.

Calculation of stride length and velocity: From the position of the sensor in space obtained after integrating the dedrifted velocity signal, the stride length $d_{stride}$ and the average stride velocity $v_{stride}$ were computed. The stride length was calculated as the L2-norm of the position in space at the index of the second midstance. Velocity was calculated by dividing stride length by stride time.

#### 2.2.5. Deep Learning

**Figure 6:**
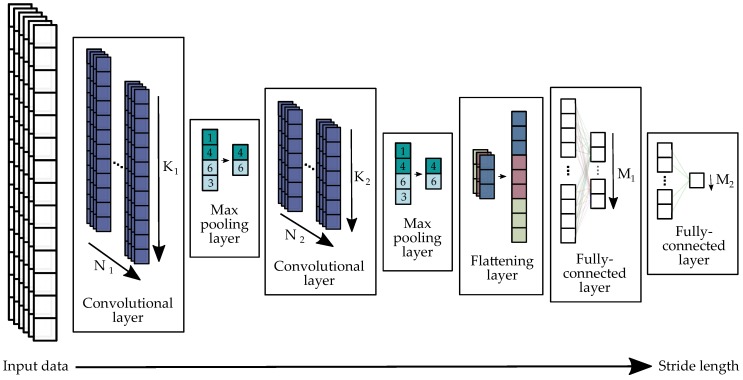
Architecture of the convolutional neural network for stride length regression based on the raw 6D-IMU signal. For the first convolutional layer, we used $N_{1}=32$ filter kernels of kernel length $K_{1}=30$. The second convolutional layer consisted of $N_{2}=16$ filter kernels of kernel length $K_{2}=15$. The first fully-connected layer had $M_{1}=128$ outputs that served as input to the second fully-connected layer, which had only a $M_{2}=1$ output. This output represented the computed stride length.

After outperforming conventional methods in various other fields like speech recognition, visual object recognition, and object detection [34], the methodology of deep learning started to become more and more popular for IMU data processing. Hannink et al. [16] introduced a deep convolutional regression network for calculating the stride length from raw IMU data in geriatric patients. The network learned a model for stride length regression based on raw IMU data without any domain knowledge. In this work, we used an adapted architecture for the stride length computation in running gait, which is depicted in Figure 6. It consisted of two convolutional layers, two max pooling layers, one flattening layer, and two fully-connected layers. For the implementation of the architecture, we used Keras [35] with a TensorFlow backend [36].

Before feeding data into the network, the segmented 6D-IMU data of a single stride were zero padded to 200 samples to assure a constant number of samples as an input to the network. One convolutional layer consisted of *N* convolution filters. The *N* outputs of a convolutional layer $O^{\left(j\right)}$ with j=1…N are called feature maps and were computed by the convolution of the six IMU input channels $x_{c}$ with c=1…6 with the filter kernel $\phi_{c}^{\left(j\right)}$ of length *K*, adding biases $b_{c}^{\left(j\right)}$ and finally applying a ReLU activation function:

(9) $$O^{\left(j\right)}=\mathrm{ReLU}\left(\sum_{c=0}^{6}\left(\Phi_{c}^{\left(j\right)}\times x_{c}+b_{c}^{\left(j\right)}\right)\right)$$

This formula has to be applied for all j=1…N filters to produce *N* feature maps $O^{\left(j\right)}$ after each convolutional layer. Thus, the two tunable parameters in the convolutional layers are the number of kernel coefficients *K* and the number of filters *N*. In the first convolutional layer, the kernel size was $K_{1}=30$ and the number of filters $N_{1}=32$. In the second convolutional layer, the kernel size was $K_{2}=15$ and the number of filters $N_{2}=16$ filters. After each convolutional layer, the resulting feature map was fed into a max pooling layer, which downsampled the resulting feature map by a downsampling factor of two by taking the maximum in non-overlapping windows of size two.

After the second max pooling layer, the feature map was flattened to produce a one-dimensional feature list that can be fed into the fully-connected layers. Thus, the flattening layer appended the $N_{2}$-dimensional output of the second max pooling layer after each other into one feature list. The two fully-connected layers at the end of the architecture computed a weighted sum of all $k=1\ldots N_{f}$ input features $\varphi_{k}$ of the one-dimensional feature vector with weights $w_{k,j}$ and added biases $b_{k}$. A ReLU function again activated the positive features.

(10) $$F_{j}=\mathrm{ReLU}\left(\sum_{k=0}^{N_{f}-1}\left(w_{k,j}\cdot \varphi_{k}+b_{k,j}\right)\right)$$

The outputs of the fully-connected layers were feature lists $F_{j}$ with j=1…M, where *M* describes the number features. In our architecture, the first fully-connected layer had $M_{1}=128$ output features. The second fully-connected layer had only $M_{2}=1$ output feature, which was the resulting target value. In our implementation, the regressed target value was the stride length.

To prevent overfitting, we also added a dropout layer to our network [37]. The dropout layer was stacked between the two fully-connected layers and dropped 30% of the neurons. During training, we fed the data into the network in five epochs with a batch size of 16. We trained the network both for the stride length and for the velocity. The network with the stride length as the output outperformed the velocity approach and was therefore used for the evaluation in this publication. Thus, the velocity $v_{stride}$ for the *Deep Learning* approach was computed by dividing the stride length $d_{stride}$ obtained from the neural network by the stride time $t_{stride}$ obtained from the stride segmentation.

### 2.3. Evaluation

#### 2.3.1. Lab Study

The results of the lab study dataset will be evaluated using the mean error (ME) and standard deviation (Std), the mean absolute percentage error (MAPE), and the mean absolute error (MAE). We provide all these measures to make our results comparable to prior studies.

For the evaluation of the *Acceleration* and *Deep Learning* algorithms, we used leave-one-subject-out cross-validation to prevent overfitted results. We also show Bland–Altman plots [38] to visualize the results.

#### 2.3.2. Field Study

For the evaluation of the 3.2-km field study dataset, we used the MAE to evaluate the total distance of the runs. After segmenting the strides and calculating the stride lengths for each stride, we accumulated the single stride lengths and compared them to the ground truth value of 3200 m. The reason for choosing the MAE for this evaluation was the fact that the absolute deviation of the ground truth value is of great importance to runners. For the *Acceleration* and *Deep Learning* algorithms, we computed the regression models based on the lab study dataset. Due to having different subjects participating in the lab and the field study, the results were not overfitted. The GPS measurements of the total distance of the individual runs were also evaluated by comparing them to the gold standard value of 3.2 km.

## 3. Results

### 3.1. Lab Study

Table 4 depicts the mean errors and standard deviations for both stride velocity and stride length of the four different algorithms for the lab study dataset. The results were averaged over all strides in the lab study dataset. The results show that the *Trajectory* algorithm performed best considering both the ME ± Std and the MAE.

**Figure 7:**
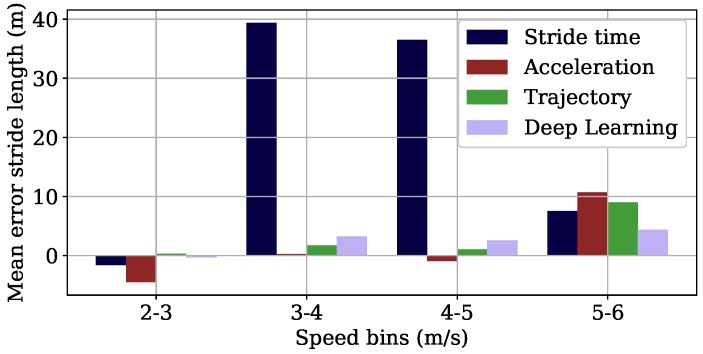
Mean error of the stride length of the four different algorithms for the different velocity ranges the subjects ran in the lab study.

Figure 7 shows the results of the stride length for the different velocity ranges. The MEs of the *Deep Learning* algorithm increased with higher velocities. The *Trajectory* showed lower MEs for the three slower velocity ranges than for the highest velocity range. The *Acceleration* algorithm showed small errors from 3–5 m/s. Its performance dropped for the outer velocity ranges from 2–3 m/s and from 5–6 m/s. The *Stride time* algorithm worked well for the velocity range of 2–3 m/s and 5–6 m/s; however, it showed large errors of more than 40 cm for the other velocity ranges.

**Figure 8:**
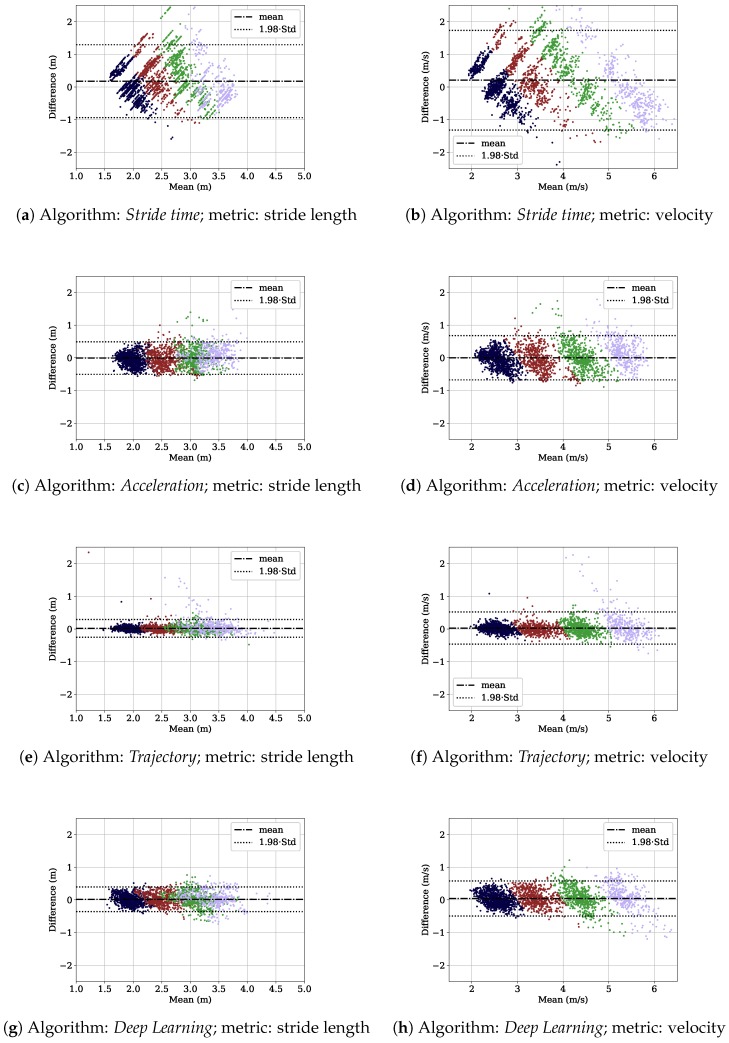
Bland–Altman plots for stride length and velocity for the four algorithms. Each row contains the metrics for one algorithm. The individual samples are color coded depending on the velocity bin of the sample: 2–3 m/s blue, 3–4 m/s red, 4–5 m/s green, 5–6 m/s purple. The dotted-dashed horizontal lines depict the mean error and the dotted horizontal line the 95% confidence interval.

Figure 8 shows the Bland–Altman plots for both the stride length and the average velocity per stride for the lab study dataset. The results are color coded into the velocity ranges presented in Table 2. The *Trajectory* algorithm performed well for velocities up to 5 m/s. For the high velocity range, larger errors could be observed. The *Stride time* algorithm performed worst and showed a linear error distribution in the Bland–Altman plots. In the *Acceleration*, *Trajectory*, and *Deep Learning* plots for stride length, we see the samples of the different velocity ranges overlapping. This overlap is not visible in the velocity plots.

### 3.2. Field Study

**Figure 9:**
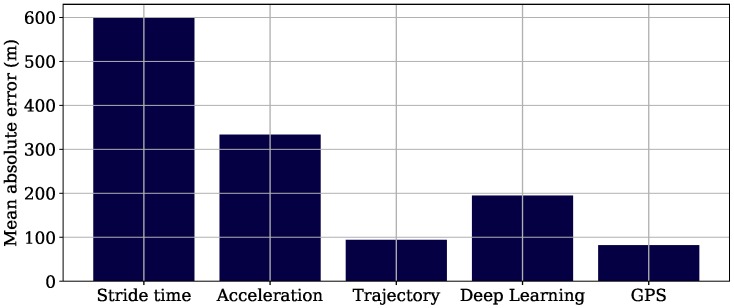
Mean absolute error of the 3.2-km run for the four different algorithms and GPS.

Figure 9 shows the MAE of the total running distance for the field study dataset, both for the algorithms and GPS-based estimation. The figure indicates that the *Trajectory* algorithm performed best with a MAE of 94.0 m. The error was comparable to that of the GPS-based estimate (82.1 m).

## 4. Discussion

Firstly, we will compare our results to existing literature. Afterwards, we will discuss the results of the lab study including a detailed evaluation of the individual algorithms with respect to their accuracy and their advantages and disadvantages in a smart shoe scenario. Special emphasis will be placed on the number of sensors that are needed to run the algorithms and the underlying power consumption of these sensors. Finally, the results of the field study on the tartan track will be discussed.

### 4.1. Comparison to Existing Literature

Different papers already evaluated the stride length or the velocity of single strides. Three of these papers are listed in Table 5. These three publications used similar approaches to ours: Bailey et al. [12] used a trajectory approach using a linear dedrifting technique; Gradl et al. [6] used the described acceleration approach; and Hannink et al. [16] a DCNN approach.

With respect to the standard deviation, the results of the trajectory implementation of Bailey et al. [12] are better than our results (Table 4). They also evaluated 1800 strides; however, these strides only originated from running velocities ranging from 2.3–3.4 m/s. We also evaluated our results for this velocity range and obtained an error of 0.004 ± 0.107 m/s. We observe that our standard deviation is still higher than the standard deviation reported from Bailey et al. One reason for that might be the higher number of different runners with different running styles who participated in our study. Furthermore, their study was conducted on a treadmill. On a treadmill, the variability of different strides at a given velocity is lower and does not reproduce overground running kinematics [39].

The errors reported by Gradl et al. [6] were obtained on a smaller database than the one presented in this paper. Thus, our worse results are due to the higher variability in our dataset, which the second degree polynomial could not appropriately approximate.

The results of Hannink et al. [16] were evaluated for gait in geriatric patients. Hence, there is a general difference in the stride patterns, causing differences in the results. Further differences between the setup of our network architecture and study population are listed and discussed in the following section.

### 4.2. Lab Study

In this section, we will discuss the results of the lab study for each algorithm in detail with respect to their advantages/disadvantages and the number of sensors needed for their implementation.

**Figure 10:**
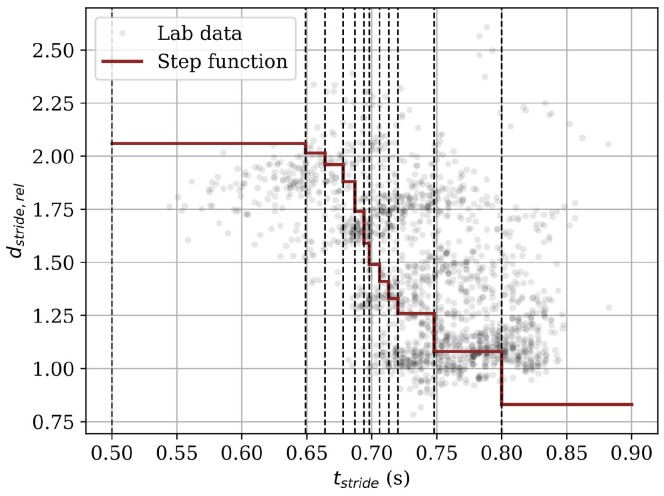
Visualization of the correlation between the stride time $t_{stride}$ and the relative stride length $d_{stride,rel}$ for male subjects. The light gray dots depict the data obtained from the field study, whereas the red curve and the black dashed lines visualize the step function obtained from literature and implemented in the *Stride time* algorithm.

**Stride time:** The *Stride time* algorithm leads to the lowest accuracy for the lab study dataset. Even though stride time and stride length relative to the subject’s height correlate non-linearly, the correlation does not seem to be high enough to compute velocity and stride length accurately. The low correlation is also visible in Figure 10. The gray dots are the relative stride length values obtained from the lab study dataset, and the red line is the step function for male subjects defined in Table 3a. We see that the step function does not approximate the underlying data accurately. The standard deviation of the relative stride length within a certain stride time range (e.g., $0.748<t_{stride}\leq 0.800$) of the step function is high. This is due to the fact that velocity is controlled by stride frequency and stride length. The *Stride time* algorithm cannot handle that fact, as it only depends on stride frequency.

In the Bland–Altman plots for the stride length metric (Figure 8), the other three algorithms showed overlapping sample clouds. This indicates that people increased their velocity both by increasing their stride length and by decreasing their stride time in higher velocities. The other algorithms are capable of dealing with this effect due to the fact that the sample clouds are separated in the Bland–Altman plots of the velocity metric. This is not observable in the plots for the *Stride time* algorithm. Thus, the other algorithms can deal better with the velocity control via stride frequency and stride length.

Furthermore, we want to discuss the shape of the *Stride time* algorithm’s Bland–Altman plots briefly. The long diagonal lines in the plots (Figure 8b) originate from the steps in the step function introduced in Table 3. One line belongs to one stride time range. The small deviations within the diagonals originate from the different body heights. We observed that for some stride time ranges, the gold standard velocity ranged from 2–6 m/s (color coded within one diagonal), showing that the stride time ranges of the step function obtained from the literature do not generalize well. Furthermore, the relative stride lengths presented in Table 3 are averaged over specific study populations. Even if a subject controls its stride frequency in the exact same manner as encoded by the stride time ranges of the step function, the resulting stride length could be incorrect due to an incorrect relative stride length.

Despite the algorithm’s low accuracy, an advantage of the stride time algorithm is that it can be implemented very energy efficiently. In the case of an IMU scenario, only a stride segmentation is necessary to compute the stride time. The stride segmentation presented in this paper only needs the sampling of the acceleration in the dorsoventral direction; thus, a 1D-accelerometer would be sufficient. In fact, strides could be segmented without an IMU using sensors such as piezo-electric switches to detect the ground contact [40].

**Acceleration:** The plot with the ME for the different velocity ranges in Figure 7 shows that the *Acceleration* algorithm works better for the the two velocity ranges from 3–4 m/s and 4–5 m/s. In addition, the Bland–Altman plots in Figure 8 show outliers especially for the highest velocity range for both the stride length and the average velocity. The reason for that can be observed in Figure 3, where we see that the second degree polynomial used to map the velocity integration value ι to the velocity value approximates the reference data better for the velocity range from 3–5 m/s and especially not well for the highest velocity range. This can be explained by the spread of the underlying data being too large to be represented by the polynomial.

However, the *Acceleration* algorithm outperforms the *Stride time* algorithm and shows comparable performance to the *Deep Learning* algorithm for the velocity range of 3–4 m/s. The advantage of the *Acceleration* algorithm over the better performing *Trajectory* and slightly better performing *Deep Learning* algorithm is its energy efficiency. For the computation of the stride length and the velocity, only a triaxial accelerometer needs to be sampled. Sampling only an accelerometer consumes less energy than sampling the gyroscope or sampling both sensors. For example, for the MPU9250 from InvenSense, the supply current needed for sampling only the accelerometer is less than 15% of the current needed for sampling both the accelerometer and the gyroscope [41]. Furthermore, the sampling rate can be further reduced for the *Acceleration* algorithm [6]. We also tested the reduction of the sampling rate for the lab study dataset and observed that a reduction to 60 Hz does not affect the accuracy of the algorithm. With such a low sampling rate, the energy consumption can be further reduced. Another advantage of the algorithm is its generalizability and its applicability to other movements like side stepping [6].

**Foot trajectory:** The *Trajectory* algorithm performs best for the lab study dataset. Especially for velocities up to 5 m/s, the algorithm achieves a ME of less than 0.012 m for the stride length and 0.014 m/s for the average velocity. For velocities higher than 5 m/s, the accuracy drops. In the Bland–Altman plots (Figure 8e,f), outliers for this velocity range are visible. The zero-velocity update based on the detection of the minimum energy in the gyroscope signal is error prone for such high velocities. The foot has no real zero-velocity phase and is always in motion. Thus, the underlying zero-velocity assumption does not hold. One way how to improve this algorithm is to propose a better solution for the initial condition when applying it to higher running velocities. Future work could evaluate whether a regression model based on the velocity during the swing phase would be a better initial condition.

**Figure 11:**
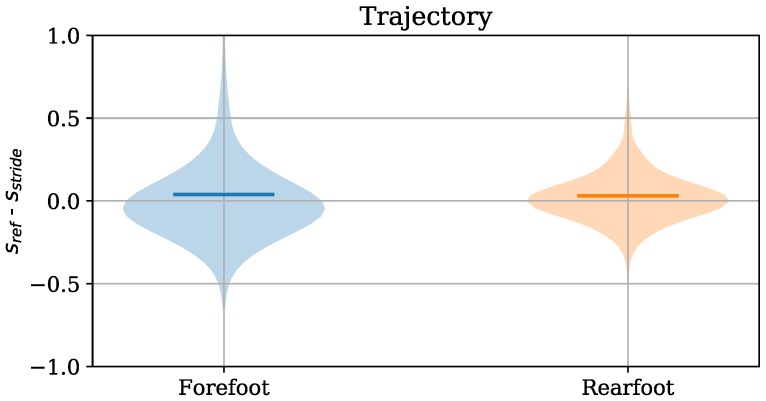
Violin plots of the error ($s_{ref}-s_{stride}$) in the velocity computation for forefoot strikers and rearfoot strikers.

For the *Trajectory* algorithm, we were also interested in the applicability of the zero velocity update for the different strike types due to the foot never being flat on the ground for forefoot runners. Hence, we also evaluated the accuracy of the *Trajectory* algorithm for the different strike types. The violin plots for forefoot and rearfoot runners are depicted in Figure 11. The plots show that the MEs do not differ significantly for the two strike types. However, the standard deviation is higher for forefoot runners. The low ME both for the forefoot and the rearfoot strike type can be explained by the fact that we align the foot during the zero velocity phase with gravity. The higher standard deviation originates in the more dynamic nature of the forefoot running style. Thus, the zero velocity phase cannot be detected accurately, which results in higher errors.

An advantage of the *Trajectory* algorithm is that it provides more information about the stride than the velocity and the stride length. During the computation of these parameters, the orientation of the shoe in space is also calculated, which allows for a determination of other parameters like the sole angle, which defines the strike pattern or the range of motion in the frontal plane that is associated with pronation [42]. Furthermore, the algorithm uses solely signal processing and has no training phase, which makes it well applicable to unseen data. This holds for lower velocities and the transition to walking.

In terms of an embedded implementation and energy efficiency, the *Trajectory* algorithm needs both accelerometer and gyroscope data. Thus, it needs more energy than the *Stride time* and the *Acceleration* algorithm for acquiring 6D-IMU data.

**Deep learning:** The *Deep Learning* algorithm produced an ME of less than 0.095 m/s for the velocity and 0.104 m for the stride length for all velocity ranges in the lab study dataset. Compared to Hannink et al. [16], we reduced both the number of filters in the second convolutional layer and the number of outputs in the fully-connected layer, because the results using the identical structure yielded worse results for our use case. The differences in the architecture are listed in Table 6. Generally, the performance of the DCNN network is worse compared to the results reported in [16].

We see that our approach needs less parameters due to the reduction of filters in the second convolutional layer and the smaller output number of the fully-connected layer. However, our results show a larger error. The reason for that might be a larger variation in our training data and the different strike pattern in running. The range of the target parameter of stride length is 3.62 m in the lab study of this work and 1.16 m in the dataset for geriatric patients of Hannink et al. [16]. The strike patterns in running differ significantly for forefoot and rearfoot runners, which also introduces more variation in the input data.

Besides, we observed that during training, the training errors and validation errors still varied after the five training epochs, even though we had more training samples than Hannink et al. [16]. Increasing the number of epochs or batches did not change the varying validation errors. This indicates that the DCNN does not generalize well. Thus, the results might be further improved by incorporating more data samples in the training process of the network.

The embedded implementation of the presented method is a challenge as the DCNN model comprises 85,425 parameters. However, it is still in a range where it can be implemented on a microcontroller. For this method, the acceleration and the gyroscope have to be sampled. This further increases the energy demand compared to the *Acceleration* approach. Taking computational effort and performance into account, the *Acceleration* method would be a better trade-off for an embedded implementation.

### 4.3. Field Study

The aim of the field study dataset was the evaluation of the estimation of the overall distance of a run in an outside and real-world scenario. The *Trajectory* algorithm also worked best for this dataset. With an MAE of 94.0 m, it is comparable to the results of GPS, which also produced an MAE of 82.1 m, and is used in state-of-the-art running platforms tracking athlete performances. Besides, the IMU technology has the advantage that it allows velocity and distance computations indoors or in scenarios where no satellite connection for GPS is available. Based on the presented results, we argue that although the *Trajectory* algorithm has high standard deviations in the lab study for the stride length calculation, these have no major impact on the computation for longer distances based on stride length. We believe this is due to errors canceling out over time. As the subjects’ average velocity was 3.48 m/s during the data acquisition, the high velocity range of 5–6 m/s was not reached for the amateur runners that participated in this study. We expect the results to be worse for the high velocity range, which can be reached by professional athletes.

The *Stride time* algorithm showed the worst performance for the field study dataset (MAE of 599.7 m). Despite its best energy efficiency, our results indicate that its accuracy is too low to use for tracking velocity and distance. The *Deep Learning* approach (MAE 194.5 m) performs better than the *Acceleration* approach (MAE 333.1 m). Due to the fact that the the neural network also needs the 6D-IMU data as an input, it has no benefit compared to the *Trajectory* approach, which performs better. The *Acceleration* approach only requires the sampling of the triaxial accelerometer, which makes it more energy efficient. Despite its decreased accuracy, we propose to use this algorithm in use cases where very strict energy limitations occur.

## 5. Conclusions and Future Work

In this study, we compared four different algorithms with respect to their performance on stride length and mean average velocity per stride calculation for running. We conducted two studies to evaluate the accuracy of the algorithms: one study in a laboratory environment with a motion capture system as the ground truth, in which we acquired 2377 strides of 27 subjects, and one field study in a real-world scenario. We showed that the *Trajectory* algorithm performs best and especially well for velocities up to 5 m/s. The results of the field study showed that this algorithm does not only work on single strides, but also on longer outdoor runs in a real-world scenario. The MAEs for this scenario showed that the trajectory is comparable to GPS measurements, which is the common method for total distance tracking in amateur running. However, the *Trajectory* algorithm is more costly energy wise due to the fact that both the acceleration and the gyroscope have to be acquired with a sampling rate of 200 Hz. When it comes to an energy-efficient use case, the *Acceleration* algorithm is a good choice, as it only needs to sample the accelerometer, and the sampling rate can be decreased to 60 Hz.

We therefore propose the implementation of the *Trajectory* algorithm for use cases with no energy limitations and the implementation of the *Acceleration* algorithm for use cases with energy restrictions.

In future work, we want to address further parameters that can be computed using inertial measurement units and other sensors located in the sole of a running shoe. Using data acquired by sensors on both feet, it is possible to perform bi-lateral analysis by combining the information of both sensors. Thus, the contribution of the individual lower limbs to the running movement can be further evaluated. Using only data from IMUs within the sole of a running shoe and the *Trajectory* algorithm, analysis regarding imbalances in stride length, stride time or orientation of the two feet can be conducted. Furthermore, other temporal parameters like flight time or stance time could be computed by adding a toe-off detection. Due to inaccuracies with the toe-off detection in running using only one IMU per foot [43], we plan to also incorporate pressure sensors for toe-off detection into the soles of a running shoe.

## Figures and Tables

**Table 1:** Anthropometric data of subjects participating in the lab study.

Parameter	Mean ± Standard Deviation
Age (years)	24.9±2.4
Shoe size (U.S.)	9.3±1.4
Height (cm)	178.6±8.0

**Table 2:** Number of trials and recorded strides per velocity range in the lab study.

Velocity Range	# of Trials	# of Strides
2–3 m/s	10	921
3–4 m/s	10	558
4–5 m/s	15	544
5–6 m/s	15	354

**Table 3:** Definition of step functions for the relative stride length $d_{stride,rel}\left[t_{stride}\right]$ for (a) male and (b) female runners for the *Stride time* algorithm.

(a) Male			(b) Female		
$t_{\mathrm{stride}}$ (s)	$d_{\mathrm{stride},\mathrm{rel}}$	Reference	$t_{\mathrm{stride}}$ (s)	$d_{\mathrm{stride},\mathrm{rel}}$	Reference
$0.800<t_{stride}$	0.830	[5,27]	$0.800<t_{stride}$	0.826	[27,28]
$0.748<t_{stride}\leq 0.800$	1.080	[27,29]	$0.735<t_{stride}\leq 0.800$	1.110	[5,27,29]
$0.720<t_{stride}\leq 0.748$	1.260	[5]	$0.720<t_{stride}\leq 0.735$	1.260	[5]
$0.713<t_{stride}\leq 0.720$	1.330	[5]	$0.704<t_{stride}\leq 0.720$	1.400	[5]
$0.706<t_{stride}\leq 0.713$	1.410	[5]	$0.667<t_{stride}\leq 0.704$	1.500	[5]
$0.698<t_{stride}\leq 0.706$	1.490	[5]	$0.607<t_{stride}\leq 0.667$	1.720	[5]
$0.694<t_{stride}\leq 0.698$	1.590	[5]	$0.578<t_{stride}\leq 0.607$	1.920	[5]
$0.687<t_{stride}\leq 0.694$	1.740	[5]	$0.500<t_{stride}\leq 0.578$	2.080	[5]
$0.678<t_{stride}\leq 0.687$	1.880	[5]	$t_{stride}\leq 0.500$	2.170	[30]
$0.664<t_{stride}\leq 0.678$	1.960	[5]			
$0.649<t_{stride}\leq 0.664$	2.015	[5]			
$0.500<t_{stride}\leq 0.649$	2.060	[5]			
$t_{stride}\leq 0.500$	2.170	[30]			

**Table 4:** Mean error (ME) and standard deviations (Std), mean percentage error (MAPE), and mean absolute error (MAE) of stride length and average velocity per stride of the four algorithms for the lab study dataset.

Parameter	Error Measure	Stride Time	Acceleration	Trajectory	Deep Learning
	ME ± Std (m/s)	0.209 ± 0.782	0.005 ± 0.350	0.028 ± 0.252	0.055 ± 0.285
Velocity	MAPE (%)	17.2	7.7	3.5	5.9
	MAE (m/s)	0.622	0.272	0.133	0.216
	ME ± Std (cm)	17.7 ± 57.3	−0.5 ± 25.6	2.00 ± 14.1	2.5 ± 20.1
Stride length	MAPE (%)	17.1	7.9	2.8	5.9
	MAE (cm)	45.2	19.9	7.6	15.3

**Table 5:** Results of other publications related to stride length and velocity calculation.

	Gait Type	# Subjects	# Strides	Parameter	Error Measure	Result
Bailey et al. [12]	Running	5	1800	Velocity	ME	0.04 ± 0.03 m/s
Gradl et al. [6]	Running	9	795	Velocity	MAPE	6.9 ± 5.5%
Hannink et al. [16]	Walking	101	∼1392	Stride length	ME	0.01 ± 5.37 cm

**Table 6:** Differences in the study setup and architecture presented in [16] from our DCNN implementation.

			# Parameters		Range Stride	# Training
	$N_{2}$	$M_{1}$	Trained	ME ± Std	Length Data Set	Samples
Hannink et al. [16]	64	1024	2,332,385	0.01 ± 5.37 cm	0.14–1.30 m	∼1392
Our approach	16	128	85,425	1.3 ± 19.4 cm	1.22–4.84 cm	2377
